# Letter to the Editor regarding lateral versus posterior quadratus lumborum block in children undergoing open orchiopexy: a double-blind randomized clinical trial

**DOI:** 10.1016/j.bjane.2025.844713

**Published:** 2025-11-21

**Authors:** Parth Aphale, Himanshu Shekhar, Shashank Dokania

**Affiliations:** Dr. D.Y. Patil Vidyapeeth (Deemed to be University), Pimpri, Pune, Maharashtra, India

Dear Editor,

We read with great interest the recent article by Mutlu et al. comparing lateral and posterior Quadratus Lumborum Block (QLB) in children undergoing orchiopexy (Mutlu ÖPZ, Kendigelen P, Tutuncu AC. Braz J Anesthesiol. 2025;75:844661). This well-conducted double-blind randomized trial addresses an important gap in pediatric regional anesthesia, given the paucity of direct head-to-head comparisons of QLB approaches in children.^1^ I commend the authors for their methodological rigor and adherence to CONSORT guidelines.

However, several aspects warrant further discussion. First, while both QLB approaches demonstrated equivalent efficacy, the study did not include a control group (e.g., caudal block or systemic analgesia), which would have contextualized the clinical advantage of QLB over more traditional techniques. Previous systematic reviews indicate that QLB reduces pain scores and opioid consumption compared to caudal blocks in children. Including such a comparator could have strengthened the translational impact of the findings.^2^

Second, the authors highlighted the limitation of not assessing the sensory block level intraoperatively. This omission makes it difficult to correlate anatomical spread with clinical outcomes. Recent imaging and cadaveric studies suggest that the extent of injectate dispersion in QLB can be highly variable. Without dermatomal mapping, it remains unclear whether inadequate coverage of scrotal innervation contributed to the requirement for rescue analgesia in nearly one-third of patients in both groups.^3^

Third, the reliance on parental reporting using Wong-Baker scores after discharge raises concerns about inter-rater reliability. Although pragmatic for outpatient surgery, this method introduces subjectivity. Combining objective pain assessment with validated observer-based tools tailored for different age groups may yield more robust data.^4^

A final consideration pertains to block selection in clinical practice. While the authors conclude that lateral QLB may be technically simpler, the choice of approach may also depend on patient positioning, anesthesiologist expertise, and potential spread patterns. It would be valuable if future studies incorporated long-term outcomes (e.g., incidence of chronic post-surgical pain) and stratified analysis by age groups, given that anatomical fascial characteristics differ significantly between infants and older children.^5^

In conclusion, Mutlu et al. provide valuable evidence that both lateral and posterior QLB yield comparable perioperative analgesia in pediatric orchiopexy. Future trials incorporating control groups, standardized sensory mapping, and extended follow-up are essential to refine the role of QLB approaches in pediatric pain management.

## Data availability statement

The datasets generated and/or analyzed during the current study are available from the corresponding author upon reasonable request.

## Funding

None.

## Authors’ contributions

Himanshu Shekhar: Conception and design; final review; conceptualization.

Parth Aphale: Data acquisition; writing the manuscript.

Shashank Dokania: Analysis; interpretation.

## Declaration of competing interest

The authors declare no conflicts of interest.
